# Identifying gaps in healthcare: a qualitative study of Ukrainian refugee experiences in the German system, uncovering differences, information and support needs

**DOI:** 10.1186/s12913-024-11052-6

**Published:** 2024-05-04

**Authors:** Kristin Rolke, Johanna Walter, Klaus Weckbecker, Eva Münster, Judith Tillmann

**Affiliations:** Institute of General Practice and Primary Care, Chair of General Practice I and Interprofessional Care, Witten/Herdecke University, Alfred-Herrhausen-Str. 50, 58448 Witten, Germany

**Keywords:** Ukraine, Health system, Refugees, Healthcare, Information needs, Displacement

## Abstract

**Background:**

The 5.8 million Ukrainian refugees arriving in European countries must navigate varying healthcare systems and different and often unknown languages in their respective host countries. To date, there has been little exploration of the experiences, perceived differences, information and support needs of these refugees regarding the use of healthcare in Germany.

**Methods:**

We conducted ten qualitative interviews with Ukrainian refugees living in Germany from February to May 2023, using Ukrainian, English and German language. The transcribed interviews were analysed using the qualitative content analysis method according to Kuckartz and Rädiker with the MAXQDA software.

**Results:**

In general, participants consistently had a positive experience of the German healthcare system, particularly regarding the quality of treatments and insurance. Differences have been reported in the structure of the healthcare systems. The Ukrainian healthcare system is divided into private and state sectors, with no mandatory insurance and frequent out-of-pocket payments. Pathways differ and tend to focus more on clinics and private doctors. General practitioners, often working in less well-equipped offices, have only recently gained prominence due to healthcare system reforms. Initiating contact with doctors is often easier, with much shorter waiting times compared to Germany. Interviewees often found the prescription requirements for many medications in Germany to be unusual. However, the mentioned differences in healthcare result in unmet information needs among the refugees, especially related to communication, navigating the healthcare system, health insurance, waiting times and medication access. These needs were often addressed through personal internet research and informal (social media) networks because of lacking official information provided during or after their arrival.

**Conclusions:**

Despite the positive experiences of Ukrainian refugees in the German healthcare system, differences in the systems and language barriers led to barriers using healthcare and information needs among refugees. The dissemination of information regarding characteristics of the German health care system is crucial for successful integration but is currently lacking.

**Trial Registration:**

German Clinical Trials Register: DRKS00030942, date of registration: 29.12.2022.

**Supplementary Information:**

The online version contains supplementary material available at 10.1186/s12913-024-11052-6.

## Background

Since the beginning of the war in Ukraine on 24th February 2022, more than 5.8 million people from Ukraine have been registered as refugees in European countries; in Germany, the number is estimated at more than one million in 2023 [1]. 80% of adult refugees in Germany are women, nearly half of them came to Germany with their minor children and live mostly in private accommodations [2]. Since June 2022, refugees from Ukraine are not required to go through an asylum procedure due to the Temporary Protection Directive (2001/55/EG), but receive temporary protection in the European Union for up to three years after registration in the Central Register of Foreigners. They are thus entitled to medical care according to the catalog of the statutory health insurance (SHI) [3]. In Germany, around 90% of the population is covered by SHI [4]. There is an obligation to be insured in a SHI up to a fixed income limit. Earners above this limit and some professional groups can opt for private health insurance. Healthcare is primarily financed by contributions from citizens and employers, as along with subsidies from tax revenue [5]. In Germany, the Standing Committee on Vaccination (STIKO) makes recommendations on the implementation of vaccinations in accordance with § 20 (2) of the Infectious Diseases Protection Act (IfSG). Vaccination is only compulsory for measles for all children aged one year and above who attend community facilities such as kindergartens or schools, as well as some occupational groups. The healthcare system in Germany is divided into outpatient care, the hospital sector and rehabilitation facilities. The general practitioner is often the first point of contact in case of health problems and refers patients to other specialists if necessary; patients can also consult other specialists directly without a referral.

Ukrainian refugees have rarely been prepared for the contact with healthcare in Germany, which can be attributed to the rapid outbreak of war and the sudden flight. German health professionals can often look back on a long history of experience in treating refugees. Nevertheless they now face new regulations due to the EU mass influx directive [3] and also a lack of information flow, e.g. in regard of information for practice teams and a lack of networking with psychotherapeutic services, contact points, medication databases and regional interpreter services [6]. Differences in the healthcare systems, such as their structure and initial contacts/pathways in case of illness, prescription rules of medication, and coping with diseases may play a role in becoming familiar with another healthcare system.

Differences in the healthcare system are rarely described in the literature or health data. Findings include, for example, corruption problems with procurement of medication [7], low vaccination coverage rates e.g. regarding polio or COVID-19, and one of the highest burdens in Europe of chronic infectious diseases such as tuberculosis and HIV in Ukraine [8, 9]. Life expectancy at birth in Ukraine is on average 65.2 years for men (Germany: 78.6 years), significantly lower than for women with 74.4 years (Germany: 83.4 years) [10, 11]. The Ukrainian healthcare system is underfunded, which leads to high out-of-pocket expenditure on the part of the population in order to achieve adequate care, although formally the healthcare system provides free care in public healthcare facilities. Besides, taking out health insurance is voluntary [12, 13].

In 2018 the state healthcare system in Ukraine was reformed. The reform of general practitioner (GP) care in Ukraine has included a free choice of doctor and stronger gatekeeping by the GP in the form of a referral system-similar to that in Germany [7, 13]. Literature from UK and Poland indicate that Ukrainian refugees are in need of healthcare services, especially for chronic diseases, gynecological and obstetric treatments as well as mental health [14, 15].

Experiences and challenges in contact with Ukrainian refugees in Germany from the viewpoint ofGPs have been researched in 2022 in a quantitative study. Communication, lack of information on previous illnesses, refugees’ expectations of services to be provided (e.g. routine unsubstantiated blood tests, thyroid tests, prescription of multivitamin supplements), and drug prescription due to unavailable or unknown medication were mentioned by GPs as the most common challenges [6]. A publication from Poland on health system differences between Ukraine and Poland indicates differences in immunization programs and prevalence of some infectious diseases [16]. However, the experiences and needs of Ukrainian refugees themselves in other healthcare systems, especially the German one, have rarely been studied and are of high current relevance. They are essential to understand patients’ points of view and to develop solutions to improve care and facilitate the arrival and integration of refugees in the German healthcare system.

That is why we focus on this topic in the following study (RefUGe-P) and aim to answer the following research questions in this publication:

How do Ukrainian refugees experience healthcare in Germany regarding major differences to the system in Ukraine and which information and support needs can be identified?

## Methods

The methodological elaboration of the study was carried out taking into account the COREQ guideline [17].

### Study design

For this study, ten Ukrainian refugees from four cities in the German region of North-Rhine-Westphalia (NRW) were interviewed in person in German, English and Ukrainian. Theoretical saturation was reached after ten interviews were conducted, so no further interview participants were recruited. The two German and two English interviews were conducted by the project staff. The six interviews in Ukrainian were conducted with the help of interpreters who translated in the interview from Ukrainian to German and vice versa. The interpreters worked on a voluntary basis, but had a lot of experience in interpreting for Ukrainian refugees at medical appointments. Additionally, information materials and consent forms were translated into the respective languages and handed out before participation.

The interview participants were recruited in various ways, mostly face-to face, via multipliers (responsible municipal employees, employees of welfare organisations, people of Ukrainian origin who volunteer to translate for refugees in the federal state of North Rhine-Westphalia) known to the project staff in four cities. The multipliers received detailed information about the planned study as well as information about the necessary inclusion criteria for participation, both orally and through a project flyer and a study information sheet. The aforementioned information materials were made available to the multipliers for their workplace and they approached refugees about the project. Interested persons were then able to contact the project staff directly or informed the multipliers about their interest for participation. The prerequisite for participation was that the interviewees were at least 18 years old and had visited a general practitioner in Germany at least once after their arrival. Additionally, it was aimed to achieve diversity among participants in terms of age, gender, family situation and health status. One potential participant was excluded because he/she had not seen a GP himself/herself as a patient.

Participants received an information sheet about the study with information on data protection and filled in a short questionnaire about sociodemographic information. These documents were also explained to every participant in person by the interviewer. All participants were fully informed by the interviewers about the study, data protection and signed written informed consent forms.

The development of the interview guide was based on the guidelines developed by Helfferich [18] and began with open narrative stimuli (opening question Appendix 1) before progressing to more specific questions. Since the research interest included different topic-specific aspects, the interview form of the problem-centered interview according to Witzel [19] was considered during guide development.

Additionally, an expert advisory board consisting of five representatives from various disciplines (including general practice, local authorities, welfare organisations and interpreters) was established to accompany the project and was involved in the preparation of the interview guide and preparation and interpretation of results. The interview guide has been newly developed for this study and is available as supplementary material (appendix 1). The main topics of the semi-structured interviews are shown there. The interview guide was pretested in two interview situations. One pretest was conducted in German, one in English whereby no adjustments had to be made.

All interviews were conducted between February and May 2023 and lasted an average of 45 min (35 min to 1 h and 10 min). They were audio-recorded and fully transcribed by an external service provider in German and English and coded in these languages. Interview protocols were written after each interview, noting special incidents and details about the interview location and atmosphere which were included in the analyses.

### Data analysis

The transcribed interviews were analysed using the qualitative content analysis method according to Kuckartz and Rädiker [20] with the computer software MAXQDA version 20 and 22. The content analysis according to Kuckartz and Rädiker can be carried out in three forms (to structure content, to evaluate it or to form types) [20]. The former was used for this project in order to be able to analyse the material in terms of content and topic.

**Fig. 1:**
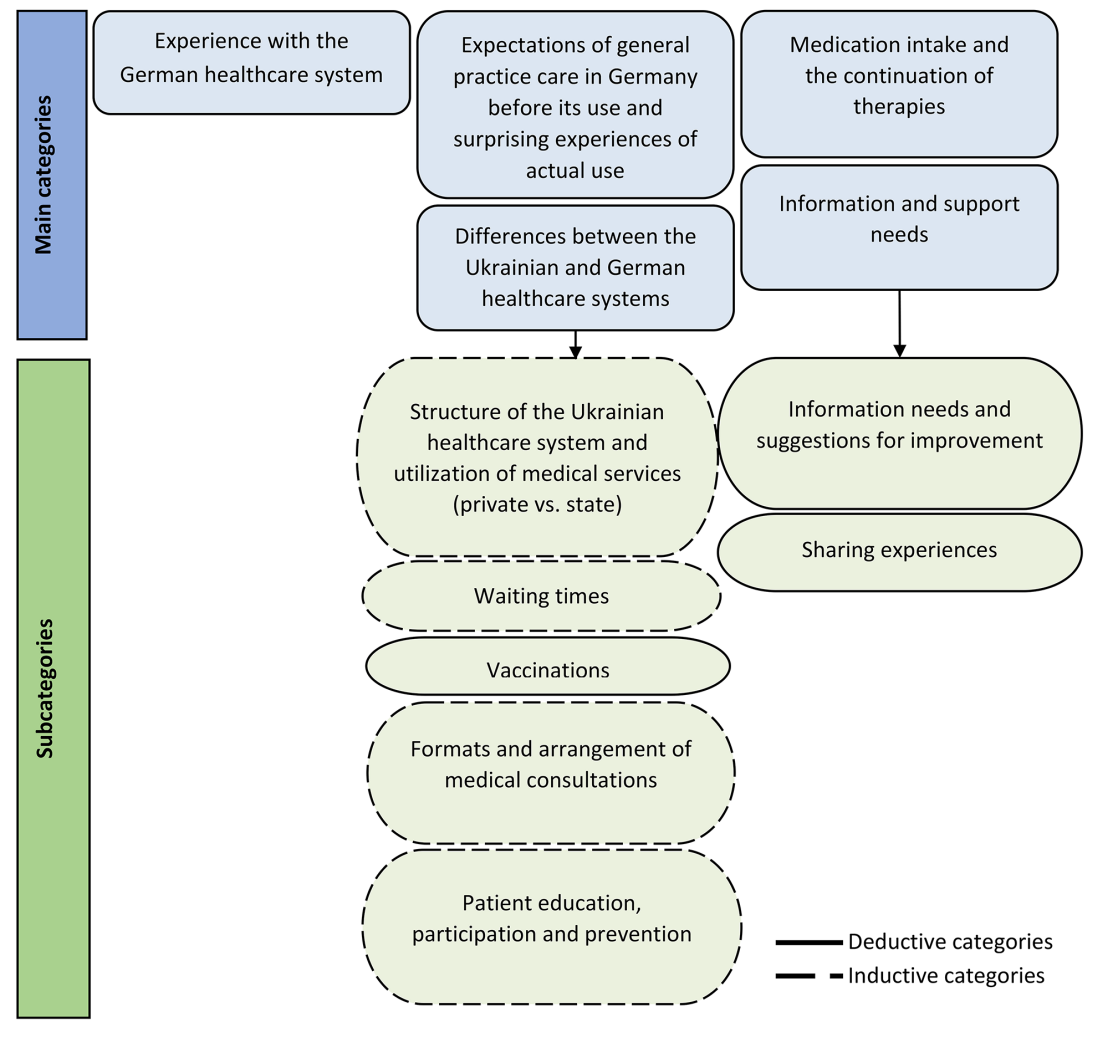
Coding tree with relevant categories for this article, RefUGe-P

The codes were developed through both deductive and inductive methods (Fig. 1). Deductive categories were formed by the subject areas that had already been recorded in the interview guide. Inductive categories were developed directly from the material.

The interviews were independently coded by two authors and the results were subsequently compared and discussed. This process aimed to improve the quality of results and mitigate the influence of subjective perspectives. One of these authors is a public health scientist with a PhD and several years of work experience in the field of migration and health and the other is a psychologist and medical student.

### Sample characteristics

A total of ten interviews were conducted with seven female and three male participants. The participants had an average age of 47.6 years (min. 29 years, max. 70 years). Additional participant characteristics are presented in Table 1.

**Table 1:** Characteristics of study participants (*n* = 10), RefUGe-P

Interview	Age	Gender	Interview language	Living in Germany since	Family situation (Living in Germany with family members or alone)	Languages spoken	Living conditions
**1**	29	Female	English	March 2022	With family members	Ukrainian, Russian, English, German	Private accommodation
**2**	32	Female	German	March 2022	With family members	Ukrainian, Russian, German	Private accommodation
**3**	70	Male	Ukrainian	April 2022	With family members	Ukrainian, Russian, Romanian	Private accommodation
**4**	70	Female	Ukrainian	March 2022	Alone	Ukrainian, Russian	Private accommodation
**5**	60	Female	Ukrainian	March 2022	Alone	Ukrainian, Russian	Private accommodation
**6**	39	Male	Ukrainian	July 2022	Alone	Ukrainian, Russian, English	Private accommodation
**7**	66	Female	Ukrainian	August 2022	Alone	Ukrainian, Russian	Institutional accommodation
**8**	40	Male	Ukrainian	April 2022	With family members	Ukrainian, Russian, English	Private accommodation
**9**	32	Female	English	March 2022	With family members	Ukrainian, Russian, English	Private accommodation
**10**	38	Female	German	March 2022	With family members	Ukrainian, Russian, English, German, Italian	Private accommodation

Refugees constitute a vulnerable group of people and this was considered during the study’s conception and research design. The interview questions were checked in advance to ensure that personal events and memories related to the flight experience were not discussed, thus minimizing the potential for retraumatization during the interviews. One interviewer was experienced in working with interview partners who had refugee experiences through prior project experience and workshops regarding this topic. The second interviewer received information and exchange of experiences via the project team. The multipliers assisting with recruitment had established positive relationships with the participants. They personally introduced the interviewers, contributing to the development of trust.

The Ethics Committee of the University Witten/Herdecke, Germany, granted approval for this study (reference number: S-219/2022).

## Results

In general, the participants’ experience of the German healthcare system was consistently positive. Interviewees positively mentioned the good and thorough treatment, as well as the extensive technical equipment of the doctors’ offices and health insurance. In particular, the inclusion of patients in treatment decisions and the information provided, for example in the case of upcoming surgical procedures, were positively emphasized in the interviews. Likewise, the reminder of the possibility to participate in preventive check-ups in old age was appreciated.

*“So the first thing that comes to my mind and I think is very good, I got the insurance and immediately got the invitations for examinations. Mammography and colonoscopy. What I have never experienced in Ukraine like this.”* (I5)

### Differences between the German and the Ukrainian healthcare system

#### Pathways and structure of the healthcare system

The interviewees reported that the healthcare system in Ukraine consists of a private and a state system, which exist side by side. Since there is no mandatory health insurance in Ukraine, according to the interviewees, citizens have the freedom to choose the services they want to use. Financial viability plays a major role in their decision-making. While many services provided by the state system are free of charge, those provided by private institutions must be paid for privately. The interviewees therefore reported different utilization patterns, which can be summarized in three scenarios: Exclusive use of the state system, exclusive use of the private system, or use of both systems.

“*I had a GP in Ukraine and then another private doctor and I communicated with both of them. Depending on the time (…) and then, because I had money, I got insurance also.*” (I5)

In case of illness, the participants in Ukraine usually went to a (poly)clinic containing many specialized medical fields, presented their concerns and were referred to the appropriate department. Some went to a physician assigned to them according to their place of residence. In urgent cases, private physicians could be visited directly on the same day. Home visits were also common in this context. Patients could request and pay for blood tests at laboratories and could immediately access their test results after processing.

*“So suppose you call and it is said that the doctor can only come the day after tomorrow, but you urgently want him to come today, then you can pay money so that he comes today. Which is also not very expensive, so the equivalent of about ten euros.”* (I6)

Some of the private institutions were considered to be of higher quality, both in medical treatment and in the equipment. Participants often had the mobile phone number of their doctors and could contact them around the clock. They got a diagnosis from remote and started treatment as suggested.

*“If I have a fever or something then I can contact my doctor conveniently via Viber or WhatsApp. I text my doctor, my daughter is sick, she can’t eat and drink and she has a fever, what should I do, and the doctor writes what I should do. For example, you have to go to the hospital now, or go to the pharmacy and buy such tablets.”* (I2)

In Ukraine a general practitioner system was also established in 2018. However, according to the interviewees, this has not yet been established everywhere across the country and private payments still exist.

### Health insurance

Interviewees reported that health insurance covering major health treatment in case of illness likewise in Germany is not common in Ukraine and just a minority can afford it. Therefore, services are used when needed, often in the private sector, in order to be treated immediately. Surgeries in particular were described as very expensive and often unaffordable. Many Ukrainians save money for years in order to pay a medical treatment. The need for regular medication and its acquisition is described to be particularly challenging for elderly people due to the low pensions.

*“(…) I don’t have this system in Ukraine because we don’t have obligatory health insurance. People in Ukraine don’t have this system. That’s why they can pay money for the first visit or for next visits and just came to the doctor and have health (…) treatment. (…) You just need to pay money and go to doctor.”* (I1)

Some of the interviewees also reported that since the reform of the healthcare system in Ukraine, health insurance with fixed monthly rates has been introduced, but that it is not functioning effectively. However, state care is often described to be of lower quality and private payments to doctors are still frequent. In Germany, they experienced the insurance system as better and medical care as more affordable.

*“Concerning the pediatrician. I can always call him and somehow ask him what medication I have to give my children now or what I should do when she feels ill. But I always send money in return. That means it’s always about money. And if you don’t do it, then nobody cares about you in the health system.”* (I7)

#### Waiting times

All of the interviewees were initially surprised by the long waiting times in Germany. Waiting for months to get specialist appointments and long waiting times in doctor’s offices and clinics were unfamiliar to the refugees. Some found these long waiting times to be stressful and problematic. Furthermore, concerns were expressed about not receiving help quickly enough in case of emergency. Interviewees also reported difficulties in finding a GP who was not busy and still accepting new patients.

*“(…) So this is long, long, long everywhere. You need to wait for an appointment, you need to wait in a waiting room. You will need to wait. Yeah, I understand. It’s different from Ukrainian system, but, yes, sometimes it’s exhausting.”* (I1)

As a result, some of the interview participants thought about returning to Ukraine for treatment or knew other people who did this.

*“She called several doctors nearby, dermatology yes, and the earliest appointment was only in three months. And what did she do? She went to Ukraine. We can go to Ukraine. And she bought a ticket in the bus, and went to city A by bus, and she did everything for one day. In the clinic she made laser, she had all blood tests. In the morning she had all the results.”* (I2)

### Prescription of medication and vaccination

The requirement for a prescription to obtain medication in Germany was unfamiliar to some interviewees. In Ukraine, various medicines were bought without a prescription, including antibiotics. The interviewees also observed that German doctors prescribe fewer medications than Ukrainian ones and they often recommend alternatives for symptoms like fever and headaches.

*“But in Ukraine it is really common (…) when my kids are sick, I always got a prescription with total list of pills, even if it is like common fever or something like that.”* (I1)

In addition to people who experienced this as positive, there were also negative statements, for example when antibiotics were not prescribed for infections and therefore then ordered in Ukraine. Additionally, individual reports highlighted differences in medication quality (better in Germany), and availability (certain Ukrainian combination medications were not available in Germany).

*“She (the GP) said to me that my daughter must drink more tea and I will be honest with you, I called to my friends. They lived in (city in Germany) and I asked them to call my doctor in Ukraine so I can find an antibiotic.”* (I9)

The mothers interviewed also wondered about the vaccinations given to their children, which they did not know from Ukraine. Most Ukrainian participants were also unaware of adult booster vaccinations. The majority was vaccinated only in childhood. Several interviewees reported that there is no structured approach to adult immunization in Ukraine. One person said that he/she did not know where to go as an adult to get vaccinations in Ukraine.

*“But what I did here, for example, the doctor here immediately offered to do so and so many vaccinations. After sixty years. And we never got such an offer in Ukraine.”* (I3)

### Information and support needs

Most of the interviewees did not receive information about the German healthcare system, medical care and insurance in Germany. Instead, they had to seek information themselves, often by doing their own research on the internet. Frequently friends, relatives, hosts, language course teachers, interpreters, etc. were asked for information. Some of the interviewees described this process as difficult. Often they asked other Ukrainians in their place of residence, e.g. through Telegram or other online Ukrainian community groups. There, doctors were recommended, lists were shared, information was spread, questions were asked and translation help was offered or requested. Interpreters are searched for through these networks as well to overcome language barriers.

*“We have some webs, there is this group in Telegram (…) and we have the big, big list and people ask maybe who knows some gynecologist or something like this and people help.”* (I9)

Table 2 specifies frequently mentioned information needs and improvement requests from refugees in Germany, along with selected quotes.

**Table 2:** Information needs and wishes of Ukrainian refugees in the German healthcare system, RefUGe-P

Thematic area	Information needs and wishes	Citations
Waiting times	• informing about long waiting times for appointments, treatments and in doctor’s offices/clinics	*“I think for Ukrainian people it’s important because they are used to another approach. They used to have help right now, right now. And for them I think it will be important to know that they need to wait, always wait. And don’t panic, just wait.”* (I1)
	• waiting times during medical emergencies	*“This time waiting for the appointments (…) if I wanted to go to the gynecologist and get an appointment and if I would be young, pregnant and only had an appointment in three months- how does that work here?”* (I5)
	• addressing and easing fears and concerns	*“And for them I think it will be important to know that they need to wait, always wait. And don’t panic, just wait.”* (I1)
Communication	• informing refugees about the requirement to attend medical consultations with an interpreter	*“Without a translator, it would be really bad. Because a lot of doctors here won’t accept anyone without a translator.”* (I8)
	• improving access to (professional) interpreters and covering costs	*“A paid interpreter would have helped. Because first of all, there aren’t very many here in city B, and if there are, they have their own lives and have to take care of other refugees here. Then it is also unpleasant to always have to ask them.“* (I8)
	• where to find interpreters	*“I tried to find (a translator) in social media groups, in social groups, where other people from Germany are who tried to help Ukrainian people.“* (I1)
	• emphasizing the importance of language courses	*“If you don’t speak the language, of course you have to find someone to help you. It can’t be an interpreter, that costs a lot of money, but many Ukrainian people complete a B1 course in a month or two, which is enough to hold a conversation and say something.“* (I2)
	• interpreters can also assist in explaining the healthcare system	*“And my idea was that someone sits in the town hall during working hours, with whom every Ukrainian can make an appointment (…) and can also explain the system better.”* (I8)
	• if possible, take medical documents with you or have them translated in Ukraine	*“And I told her that she should take all the documents and all the medical documents if she has problems with health. And if it is possible, she should have the vaccination certificate translated in Ukraine, because it is faster and cheaper than here, because she also has a daughter. And at school they need this.“* (I2)
Pathways in the healthcare system	• explaining the general practitioner system, where the general practitioner (GP) serves as the primary point of contact	*“And the doctor says no, no, you’ve come to the wrong doctor, please go to your GP. Then we first have to find out who the GP is (…)”* (I5)
	• referral system from GPs to other specialists	*“But that was actually strange to me, that if I wanted to go to the orthopedist, I should still go to the GP, even though I knew that I wanted to go to the orthopedist.”* (I5)
	• guidance on how to locate doctors in Germany	*“We have some webs, there is this group in Telegram I think. Yes, Ukrainian-her name is Ukrainian people in City X or something like this and we have the big, big list and people ask maybe who knows some gynecologist or something like this and people help.”* (I9)
	• how to get medical appointments	*“Personally, I would have been interested in how I could get to an orthopaedist, for example? What is needed for that? Or to a doctor who examines veins.”* (I4)
Health insurance	• providing information on the German health insurance system, including its benefits	*“So now I know because I’ve experienced it myself, the first, very first question is insurance. (…)The person can’t just turn up and say I want to see a doctor. It has to be via registration and insurance. Because only when you are insured you can get help from anyone, medical help.”* (I4)
	• how to get access, an insurance card, who to turn to	*“ So for me personally it (the membership to a an insurance company) took so long because when I applied I was still doing a mini-job at the time. And I thought that I had something to do with the JobCentre and I registered with the JobCentre even though I was a pensioner.“* (I4)
	• understanding what costs and health services are covered by insurance	*“(…) what the insurance will cover. So whenever I go to the doctor, the question is always “Yes, does the insurance cover it or doesn’t it cover it?” And I don’t know who the contact person would be (…)”* (I6)
Medication supply	• high-quality medications in Germany with contents matching package claims	*“And that the drugs here are good and that a lot is covered by insurance. Because in Ukraine, people are prescribed drugs that don’t work at all. Because they are poorly manufactured. And that is not the case here.”* (I7)
	• medication intake is often assessed for necessity beforehand, e.g. in case of antibiotics	*“So it was like this, I felt like I had eye problems (…) And I was first assigned a machine to measure my blood pressure during the day. And only then was I given the medication I had to take.”* (I5)

## Discussion

Our study identified perceived differences between the German and Ukrainian healthcare systems with the Ukrainian system still being shaped by out-of-pocket payments, private care, no mandatory insurance, and a GP system only gaining prominence in the recent years before the war. Easier contact to doctors with shorter waiting times and less prescription requirements for some medications have been reported about Ukraine. These differences in combination with lacking official information provided during or after arrival lead to unmet information and support needs among Ukrainian refugees living in Germany.

The results are particularly relevant in light of the fact that many Ukrainians would like to live in Germany in the long term, recorded in current surveys [2]. Therefore, it is crucial to ensure the successful integration of this patient group and a mutual understanding of their needs to provide equal healthcare opportunities. Some of the results of this article may also be of interest to other countries, as the findings on the structure and characteristics of the Ukrainian healthcare sector can be compared regionally. In addition, the results show parallels to studies on refugees from other countries of origin in Germany, e.g. persisting communication problems [21, 22].

### Pathways in the healthcare system and waiting times

In Ukraine, fast access to healthcare, especially if the services are privately paid for, results in short waiting times and easy access to doctors. Therefore, Ukrainians in the German system are not used to experience long waiting times and not to have the ability to expedite the processes themselves. As reported from the interviewees in Ukraine, patients often commission, pay for and receive their own medical analyses. In the interviews it became clear that the different approach and the different circumstances in Germany can cause a feeling of loss of control and unpredictability. As patients in Ukraine, they were able to act in a self-determined manner and, for example, pay money in order to be treated more quickly. In Germany, faster access to care is particularly important in the case of acute or life-threatening conditions. It might be perceived as impatience or high expectations, but it primarily stems from differences in healthcare systems, habits and the lack of information dissemination. Ukrainian-language information on the German healthcare system should be provided to the refugees as soon as they arrive.

### Prescription of medication and prevention

The respondents were often unfamiliar with preventive care services and booster vaccinations in adulthood. This lack of awareness may be attributed to the low vaccination rates in Ukraine prior to the war, which led to outbreaks of vaccine-preventable diseases such as measles and polio between 2017 and 2020 [23]. Despite a national vaccination schedule provided by the Ministry of health in Ukraine [24], vaccination rates are among the lowest in Europe. Preventive measures in Germany (e.g. cancer screening) were welcomed after information and explanation in our study. At the same time, people with a migration background have an on average lower level of health literacy with regard to preventive care services in Germany compared to people without a migration background [25]. Therefore, it can be useful to inform and educate patients about this approach to healthcare and to increase health literacy in general.

Participants also reported differences about how medication is prescribed and taken like quick prescription of medications by Ukrainian doctors. Since antibiotics were also sold over-the-counter in Ukraine until recently [26], self-medication occurred frequently. Having a large number of different medications were not rare in households. This old practice was criticized among some younger respondents and they appreciate the new reform regulations in Ukraine and feel comfortable with the treatment approach in Germany. It is essential for doctors and medical staff to be aware of these differences to address misconceptions and raise awareness about (in)effectiveness of medications.

### Health insurance

Participants valued their membership in the German SHI as it provided them with a sense of security. However, not all participants were accustomed to this, as they often had to save up the required amount for healthcare. Because of this difference, the respondents wished to receive more information about the scope of medical services provided by the SHI, as it was not clear to them that most costs of necessary medication are included, whilst dental treatment, for example, partially requires private payment. This information should be made available upon arrival in Germany.

### Information and support needs

None of our interviewees received information about the German healthcare system or healthcare in general through official channels. The fact that information about the health care system is often obtained via informal channels is also reported in other (inter-)national studies [22, 27]. This should be changed urgently to improve care and facilitate access for the refugees. As there is a lot of Ukrainian and Russian information online created for example by the Federal Office for Migration and Refugees (www.germany4ukraine.de), but it does not seem to reach the refugees, the distribution should be improved. This should already be done upon arrival, e.g. at registration at the Foreigners’ Registration Office or at the Citizens’ Registration Office in case of residence registration, but can also be useful in doctor’s offices.

As also identified in other studies [6, 16, 28], refugees in our study perceived some information and support needs to healthcare regarding communication. In many cases, the treating physicians demanded that an interpreting person must be present. Refugees, on the one hand, are therefore under pressure to find interpreters, who are often rare, in a country and system they often do not know and on the other hand, they always have to seek help. This situation also led them to bypass the issue and seek out Russian-speaking doctors. Still, the costs for professional interpreters are generally not reimbursed in GP practices and have to be paid by the patient [29]. There is a need for interpreters, preferably paid and professional, both when making and taking advantage of appointments. This has already been demanded for general practice and practices in general [30–32]. Biddle et al. [21] also emphasize the expansion of high-quality interpreting services in Germany. It cannot be the task of the refugees to look for and pay for interpreters. This should be urgently organized by the state, for example through (municipal) contact points for interpreter seekers and (telephone or video) interpreter services for medical consultations.

### Limitations

Around half of the interviews were conducted with the help of interpreters. The real-time translation by interpreters demands a high level of concentration. An exact reproduction of all interview content is hardly possible, so that a loss of information cannot be prevented [33]. The interpreters were known to the patients from previous medical appointments and had already established a relationship of trust with them. However, multipliers, researchers and interpreters involved made it clear that participation was voluntary and emphasised the aims of the research work. Furthermore, the interviews were solely conducted in NRW, Germany’s most populous federal state. The study could therefore be expanded throughout Germany and with more participants in order to gain further insights - including into regional differences. However, through the broad spread of age groups, gender, locations, diseases and the inclusion of several multipliers, we have attempted to get a broad picture.

## Conclusions

This study provides important and new information about the healthcare experiences of Ukrainian refugees in Germany, differences in the healthcare systems and resulting information and support needs from the perspective of refugees.

Participants’ experiences of the German healthcare system were predominantly positive, especially because of the quality of treatments and health insurance. Nonetheless, health system differences in pathways, responsibilities, structure, insurance and costs, quality, medication and prevention as well as waiting times are noticeable for Ukrainian refugees in the German healthcare system, and influence their utilization of services. On top of this, the language barrier is a huge and still unsolved problem. Disseminating information about the new healthcare system shortly after the arrival of Ukrainian refugees in Germany, conducting educational efforts and tackling language barriers are essential for successful integration, but are lacking in Germany.

### Electronic supplementary material

Below is the link to the electronic supplementary material.

Supplementary Material 1

## Data Availability

No datasets were generated or analysed during the current study.

## Abbreviations

GP: general practitioner
NRW: North Rhine-Westphalia
SHI: statutory health insurance
