# Supplementary material for: Identifying gaps in healthcare: a qualitative study of Ukrainian refugee experiences in the German system, uncovering differences, information and support needs
Source: BMC Health Serv Res. 2024 May 4;24:585. doi: 10.1186/s12913-024-11052-6 (PMC11069252; doi:10.1186/s12913-024-11052-6)
Supplement: Supplementary file 1 — Supplementary Material 1 [file 12913_2024_11052_MOESM1_ESM.docx]

**Appendix 1**

**Interview guide (only main questions)**

I would like to speak to you about your experiences with the German healthcare system, especially regarding interactions with general practitioners. I would like to hear about your experiences. You can decide what you find important and what you are willing to talk about. I will listen and take notes while you talk, in order to be able to ask possible follow up questions.

- **Opening question:** What kind of experiences have you had with the German healthcare system in general?
- **General Practice:**
- You have already contacted a general practitioner. Tell me about one of your visits to the doctor (general practitioner), starting with when you realized that you were feeling unwell or when you realized that you needed medical care and wanted to see a doctor about it.
- How did you choose your current general practitioner?
- **Barriers and supporting factors:**
- If you think back to your last visit to the general practitioner’s or doctor’s office: What was good/helpful? What was difficult/What bothered you? How did you feel?
- How did you experience communication with the doctors?
- (In the case of known/pre-existing medical conditions): How did you continue your medication?
- **Doctor-patient relationship and healthcare system in Ukraine:**
- What are important factors for you to build a trusting relationship with your general practitioner?
- What expectations do you have regarding the care provided by general practitioners in Germany?
- What kind of differences are you experiencing in comparison to the healthcare system in Ukraine?
- General practitioners in Germany are also responsible for vaccinations. How are vaccinations organized in the Ukraine?
- **Need for information and support**
- What kind of information about the (general practitioner) healthcare system in Germany did you receive before your visit to the doctor here in Germany?
- What kind of information would you have preferred to receive?
- What kind of information and/or support would you like to receive at this point?
- **Wishes and/or concrete ideas for improvement**
- What would you personally desire concerning healthcare in Germany?
- What advice would you give to other Ukrainians regarding healthcare in Germany?
- **Closing**
- Is there anything else on the topic that you consider important and would like to say about the topic?
